# Beyond personal space: Unveiling the transmission pattern of the human gut and oral microbiome

**DOI:** 10.1002/imt2.98

**Published:** 2023-03-22

**Authors:** Sergio Andreu‐Sánchez, Jiafei Wu, Jingyuan Fu

**Affiliations:** ^1^ Department of Genetics University of Groningen and University Medical Center Groningen Groningen The Netherlands; ^2^ Department of Pediatrics University of Groningen and University Medical Center Groningen Groningen The Netherlands

## Abstract

Valles‐Colomer et al. evaluated the rate of microbial strain sharing to infer person‐to‐person microbial transmission. The presence of shared strains does not necessarily indicate direct transmission between individuals. Future research should investigate the potential for noncommunicable diseases to be transmitted through microbial strain transmission. Identification of harmful strains with high transmissibility and transmission routes can enable the development of targeted interventions.

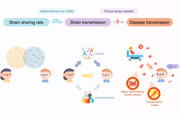

The human body is home to a remarkably diverse bacterial community, yet our understanding of how these microbes are acquired throughout life remains limited. Uncovering the origins of the microorganisms inhabiting the human body is a fundamental question in microbiome research. Previous studies have shown that the majority of microorganisms present in our bodies are acquired from other individuals [1]. During the early stages of life, infants inherit microbes vertically from their mothers, establishing a lifelong host–microbe symbiosis that plays a critical role in immune and cognitive development [2, 3]. Later in life, social interactions and shared living spaces significantly influence the composition of the human microbiome [4]. However, our current knowledge of commensal microbe transmission across hosts is limited by the absence of a large‐scale, strain‐level study of multiethnic cohorts across different geographic regions.

The recent hallmark work by Valles‐Colomer et al. [5] published in *Nature* is, to date, the largest and most comprehensive study looking at microbial transmission between individuals and populations. In total, the study gathered data from over 800 bacterial species across over 9700 samples from 20 countries, amalgamating both publicly available and newly generated metagenomics data. It assessed the strain‐level sharing rates of mother–offspring pairs, twins, families, cohabiting individuals, and individuals within a population, as well as those between different populations, providing a global snapshot of the transmission landscape of human gut and oral microbiome (Figure 1).

## STRAIN DEFINITION AND PROFILING

Assessing microbial transmission is a challenging task that requires strict tracking of the well‐defined “strains” that make up a species to understand where new colonizers originate and how long they persist. While the definition of a “strain” is disputed in the microbiome field [6], it is commonly defined as a subspecies entity with specific genetic variability. Valles‐Colomer and colleagues [7] addressed this technical issue by identifying marker genes in 646 gut and 252 oral bacterial species and constructing phylogenetic trees of the majority of strains using StrainPhlan4. The authors defined the strain boundary using a normalized phylogenetic distance threshold that could distinguish strains detected in the same individual at different time points from unrelated samples. Although this definition was optimized for specific populations and evolutionary times (longitudinal samples were taken at most 6 months apart), it appears to be a stable and reliable metric that the authors then used to explore consistently observed patterns, independent of cohort, population and technical differences between studies.

**Figure 1:**
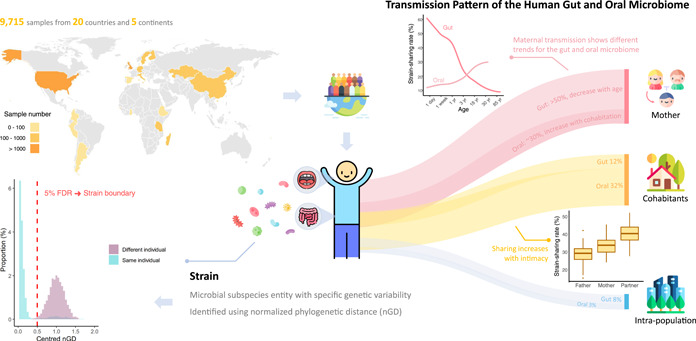
Summary of Valles‐Colomer and colleagues' study. The study includes 9715 samples from 20 countries and 5 continents, providing a global snapshot of the transmission landscape of human gut and oral microbiomes. Strains are defined using species‐specific normalized phylogenetic distance (nGD) thresholds, which best separated same‐individual longitudinal samples from unrelated individual samples. Strain transmission occurs in both the gut and oral microbiome. Individuals share strains with their mother, their cohabitants and other individuals in the same population. Maternal transmission showed different trends in the gut and oral microbiome. Infants share >50% of gut microbial strains with their mothers, with the sharing rate decreasing with age as new strains are acquired from external sources. Oral microbial sharing between mother and child is low in early childhood, but increases with cohabitation duration. Among individuals and cohabitants, oral microbial sharing increases with intimacy, with partners sharing at a higher rate than mother‐offspring pairs. The icons used in the image are partially sourced from flaticon.com.

## TRANSMISSION OF GUT AND ORAL MICROBIOMES

The authors observe that the transmission of both gut and oral microbiomes is common and largely driven by close contact and social interactions.

Mother–infant pairs typically shared the most strains (median 34%), with vertical transmission stabilizing after 3 years of age, leading to a level of sharedness that persists for a long time. Although sharedness tended to decline with age as individuals acquired new strains from other sources later in life, a high level of mother–offspring sharedness was still observable at older ages and without cohabitation, possibly due to early‐life imprinting or common social interactions. The observation that adult twins who no longer cohabit also share strains (median 8%) suggests that this early‐life imprinting plays a role, although common social interactions cannot be ruled out as a contributing factor.

Individuals living in the same household exhibited high rates of strain sharing, with significant variation across households (between 11% and 71%, with a median of 12%). Although variable, these sharing patterns seem to be consistent across different geographical locations and lifestyles. Individuals from the same village also exhibited strain sharing (median 8%), although this rate was much lower than that shared by members of the same household. The intrapopulation transmission was heterogeneous across populations and was influenced by population characteristics.

In contrast to the gut microbiome, oral bacteria sharedness was even more frequent and mostly horizontally acquired, with saliva being a direct transmission vehicle. While maternal transmission at birth is minimal, the number of shared bacteria increased as individuals spent more time together, resulting in higher oral strain‐sharing rates in cohabiting individuals (median 32%) compared to noncohabiting individuals in the same population (median 3%) and different populations (median 0%).

Finally, the authors were able to observe some common trends in the bacteria that are shared, with Gram‐negative bacteria transmitting better in households and mother–child pairs and spore‐forming bacteria and fecal aerotolerant bacteria transmitting better within populations.

## FUTURE PERSPECTIVES

Valles‐Colomer and colleagues have provided a comprehensive assessment of gut and oral microbiome transmission across diverse populations that lays a crucial foundation for future research in the field. While this work did include data from multiple countries and continents, further inclusion of understudied populations and exploration of additional microbial species will be necessary to generalize the trends they describe.

Valles‐Colomer and colleagues' work has discerned some common characteristics of strains that exhibit a propensity for transmission, but many questions remain to be answered. Strain transmission may be a random process across social interactions, but strain engraftment or colonization after transmission can vary greatly, as has been observed in fecal microbiota transplantation [8]. Future research should focus on elucidating factors that may impact the transmissibility of a strain, including strain‐specific functional capabilities such as antibiotic resistance or virulence factors, as well as host‐related factors such as differences in the microbial composition of the strain recipient. Moreover, although the study reasonably assumes that individuals within the same population or household have comparable lifestyles, future research should strive to collect more extensive and detailed information on environmental factors, especially those associated with diseases.

It is expected that ongoing developments in the microbiome field, such as deeper sequencing depth, high‐fidelity long‐read sequencing, and development of accurate strain deconvolution tools, will enable the analysis of not only the major strain in each study participant—as was assessed in Valles‐Colomer and colleagues' work—but also other minority strains. New methods that capture the proportion of each strain shared between individuals may influence the observed strain‐sharing events and could potentially lead to an updated definition of the strain‐sharing rate. These methods may also shed light on strain dynamics within a single individual followed over time and on strain evolution during transmission. The currently defined strain replacements might be due to community shifts, where both strains might coexist, and not directly attributable to a complete strain loss. Furthermore, sharedness does not always imply person‐to‐person transmission. Although the present study controlled for coacquisition from dietary sources, individuals may still acquire the same strains separately from their social networks or environment. More research is needed to analyze the impact of the surrounding relatedness or similarities. To truly assess person‐to‐person transmission, the use of species‐specific molecular clocks to estimate the evolutionary rate and timescale would be extremely powerful, providing a better understanding of how frequently strain transmission events occur and whether the transmission is one‐to‐one, one‐to‐many, or possibly obtained from an unknown third party. For instance, due to increasing globalization, foreign tourism and commerce are becoming prevalent. International travelers may acquire particular strains and bring them back to their home country, where those strains can be transmitted to other people through social interactions. Analyzing the molecular clocks of longitudinal samples of foreign visitors and their close associates can reveal the person‐to‐person transmission route. Ultimately, this type of study can lead to a more nuanced understanding of the complex dynamics underlying microbial transmission and evolution.

## IMPLICATIONS FOR HUMAN HEALTH

Human diseases are often classified as communicable and noncommunicable. Communicable diseases, also known as infectious diseases, can be transmitted from one person to another or from animals or the environment to humans. Noncommunicable diseases are not spread between individuals and include most chronic disorders such as cardiovascular disease, depression, cancer, and diabetes. Recent research has suggested that the microbiome may have a significant impact on the development of these latter conditions. Estimating to what extent microbiota can be transmitted through early‐life mother‐to‐baby and later‐life person‐to‐person transmission may challenge our definition of noncommunicable diseases.

The findings of this study highlight the widespread microbial transmission and bring attention to the potentially transmissible nature of gut/oral microbial‐associated diseases, which might have been overlooked in the context of noncommunicable diseases. Classic disease heritability estimates based on family pedigrees are often higher than the amount of variability accounted for by common human genetic variants [9], and this “missing heritability” problem might be partially mediated by shared microbiota.

Several microbial transmission routes have been suggested, with the gut‐food‐oral‐gut route appearing to be the prevalent scenario. If illness risks can be spread via microbial transmission, then appropriate measures need to be taken to prevent microbial transmission. To address this issue, surveillance programs that monitor food sources from stores and restaurants could be effective in preventing disease‐associated microbes from being transmitted. This is particularly important for high‐risk individuals and populations, where transmission is more likely, and where effective screening and surveillance programs are essential. On the other hand, upon identification of strains linked to disease traits that are liable to transmission, novel targeted interventions might be formulated to thwart transmissibility or only allow beneficial strains to be transmitted, which might be of special interest in current cohousing practices in psychiatric and geriatric communities.

The field of human microbiome research still has much to reveal, including the extent to which microbiota affects health, how microbial communities are acquired and assembled, and how we can adjust lifestyles and clinical interventions to promote the development of healthier communities. By identifying the extent and patterns of microbiome transmission between individuals, we can better understand the role of the microbiome in the development of disease and potentially develop interventions to improve health outcomes. Valles‐Colomer and colleagues' work represents an important early step on the path toward answering these questions.

## AUTHOR CONTRIBUTIONS

The manuscript was drafted by Sergio Andreu‐Sánchez and Jiafei Wu under the supervision of Jingyuan Fu. Jiafei Wu was responsible for creating the figure. All authors (Sergio Andreu‐Sánchez, Jiafei Wu, and Jingyuan Fu) participated in the critical revision of the manuscript and provided their approval.

## CONFLICT OF INTEREST STATEMENT

The authors declare no conflict of interest.

## Data Availability

This paper did not generate any new data. Please refer to the original article [5] for all data referenced in this paper.
